# Transportan 10 improves the anticancer activity of cisplatin

**DOI:** 10.1007/s00210-016-1219-5

**Published:** 2016-02-22

**Authors:** Rusiecka Izabela, Ruczyński Jarosław, Alenowicz Magdalena, Rekowski Piotr, Kocić Ivan

**Affiliations:** Department of Pharmacology, Medical University of Gdańsk, Gdańsk, Poland; Department of Chemistry, University of Gdańsk, Gdańsk, Poland

**Keywords:** Cell-penetrating peptides (CPPs), Transportan 10 (TP10), HEL299 cell line, HeLa cell line, HEK293 cell line, OS143B cell line, Click chemistry

## Abstract

The aim of this paper was to examine whether cell-penetrating peptides (CPPs) such as transportan 10 (TP10) or protein transduction domain (PTD4) may improve the anticancer activity of cisplatin (cPt). The complexes of TP10 or PTD4 with cPt were used in the experiments. They were carried out on two non-cancer (HEK293 (human embryonic kidney) and HEL299 (human embryo lung)) and two cancer (HeLa (human cervical cancer) and OS143B (human osteosarcoma 143B)) cell lines. Both complexes were tested (MTT assay) with respect to their anticancer or cytotoxic actions. TAMRA (fluorescent dye)-stained preparations were visualized in a fluorescence microscope. The long-term effect of TP10 + cPt and its components on non-cancer and cancer cell lines was observed in inverted phase contrast microscopy. In the MTT test (cell viability assay), the complex of TP10 + cPt produced a more potent effect on the cancer cell lines (HeLa, OS143B) in comparison to that observed after separate treatment with TP10 or cPt. At the same time, the action of the complex and its components was rather small on non-cancer cell lines. On the other hand, a complex of another CPP with cPt, i.e., PTD4 + cPt, was without a significant effect on the cancer cell line (OS143B). The images of the fluorescent microscopy showed TAMRA-TP10 or TAMRA-TP10 + cPt in the interior of the HeLa cells. In the case of TAMRA-PTD4 or TAMRA-PTD4 + cPt, only the first compound was found inside the cancer cell line. In contrast, none of the tested compounds gained access to the interior of the non-cancer cells (HEK293, HEL299). Long-term incubation with the TP10 + cPt (estimated by inverted phase contrast microscopy) lead to an enhanced action of the complex on cell viability (decrease in the number of cells and change in their morphology) as compared with that produced by each single agent. With regard to the tested CPPs, only TP10 improved the anticancer activity of cisplatin if both compounds were used in the form of a complex. Additionally, the complex was relatively safe for non-cancer cells. What is more, TP10 also produced an anticancer effect on HeLa and OS143B cell lines.

## Introduction

Efficient delivery of therapeutic molecules to cells is a great challenge in modern medicine and pharmacology. Recently, cell-penetrating peptides (CPPs) have received great attention as efficient cellular delivery vectors due to their intrinsic ability to enter cells and mediate uptake of a wide range of macro- or nanomolecular cargos. Generally, CPPs are relatively short cationic peptides which are classified into two groups based on their physicochemical characteristics: amphiphatic and nonamphiphatic. Among the amphiphatic are transportans (TPs) with transportan 10 (TP10) being the one of the most widely explored. CPPs are associated with cargos via covalent bonds or through non-covalent interactions. A large range of chemical agents can be regarded as cargos, i.e., plasmids, DNA, siRNA, proteins, peptides, low molecular weight drugs, and nanoparticles.

The mechanisms by which CPPs are transclocated across the biological membranes still remain unclear. It is known, however, that they involve rather an energy-independent cellular process in which different endocytotic and non-endocytotic routes are used. Which of them a CPP will utilize depends on many factors, including, among others, the cargo and the cell type it will enter (Lindgren and Langel 2011).

A large number of preclinical studies have reported on successful delivery of therapeutic cargos by CPPs in different kinds of diseases as, e.g., viral and bacterial infections, cardiology, muscular dystrophy, and cancer (Copolovici et al. 2014; Montrose et al. 2013; Mohandessi et al. 2012; Freire et al. 2013). Additionally, CPPs have also been applied in different kinds of gene modulation (Baoum et al. 2012; Kanemaru et al. 2011), and some of them demonstrated antitumor (delivery of siRNA into tumor cells) (Fang et al. 2013; Xu et al. 2013) or antiviral activities (Zhang et al. 2008).

The potential role of CPPs, as carriers for different molecules, including drugs is still a matter of considerable interest (Kocić et al. 2011), particularly in cancer therapy. Their use may improve chemotherapeutic strategies for example such as prevention of the drug resistance evolution, increase in the ability of recognition of cancer cells (targeted therapy), and enhancement of the therapeutic response to the cargo. CPPs may use as cargos anticancer chemotherapeutic drugs of small molecular weight, including doxorubicin, methotrexate, and paclitaxel (Rousselle et al. 2000; Lindgren et al. 2006; Stewart et al. 2008). These results were an incentive for the research undertaken in this study, the purpose of which was to find out whether another anticancer drug, i.e., cisplatin, may be included into the list of the abovementioned cargos.

Cisplatin (an old generation antitumor compound), being on one hand highly effective in treatment of versatile solid tumors, shows on the other severe toxicity which is a principle factor that limits its usage in clinical oncology. These limitations have motivated an extensive research effort towards development of new strategies for improving platinum therapy. One of them was introduction on the pharmaceutical market two less toxic derivatives of cisplatin, such as carboplatin and oxaliplatin. Nevertheless, its safety profile has not been satisfactory. Recently, new nanotechnology has been employed which includes among others nanocarriers for delivery of platin compounds (Oberoi et al. 2013).

Another approach to the issue of diminishing cisplatin’s toxicity is presented in this study. Namely, two CPPs with cisplatin were investigated. The chosen CPPs were TP10 (one of the transportans) and protein transduction domain (PTD4) because they fulfilled mostly the assumed criteria (chemical structure, intracellular cleavage, antitumor activity, lack of inhibition of GTP activity, and former experience at this laboratory) (Ruczyński et al. 2002). Therefore, it was interesting to find out whether the tested CPPs in the form of non-covalent complexes with cisplatin (cPt) increase the anticancer action of this drug.

## Materials and Methods

### Synthesis of TP10, PTD4, and their alkyne functionalized analogs [Lys^7^(Prop)]TP10 and Prop-PTD4

All peptides (Table 1) were synthesized by a solid phase peptide synthesis (SPPS) with the use of a Labortec AG model SP 650 peptide synthesizer and Fmoc strategy (Chan and White 2000; Ruczyński et al. 2002). TentaGel S RAM resin (capacity 0.25 mmol/g) was used as the starting material. All amino acids were coupled as active derivatives in a threefold molar excess with the use of the TBTU with addition of HOBt (1:1) in the DMF/NMP solution. Deprotection of the Fmoc group was carried out with 20 % piperidine in DMF. In case of TP10 analog, a hydrazine-labile ivDde group was used to protect the ε-amino function group of Lys^7^ residue instead of the standard acid-labile Boc group. As hydrazine removes the Fmoc group, the *N*-terminal Ala residue was protected by the Boc group. After completion of the peptide backbone synthesis, the ivDde group was removed with 10 % hydrazine monohydrate in DMF. The alkyne group (Prop) was attached to the ε-amino function group of Lys^7^ residue (in case of [Lys^7^(Prop)]TP10) or the *N*-terminal amino group (in case of Prop-PTD4) with the use of a 10-fold molar excess of propiolic anhydride. Peptides were cleaved from resins with TFA/phenol/TIPS/water (88:5:2:5) mixture, and this process lasted for 2 h. Next, crude peptides were purified and analyzed with the use of RP-HPLC, and finally characterized by MALDI-TOF mass spectrometry.

**Table 1:** Amino acid sequence of the synthesized peptides

Peptide	Amino acid sequence
TP10	AGYLLGKINLKALAALAKKIL*-NH*_*2*_
TAMRA-TP10 [Lys^7^(C(O)-Tra(1,4)-PEG_3_-TAMRA)]TP10	AGYLLGK(C(O)-Tra(1,4)-PEG_3_-TAMRA)INLKALAALAKKIL*-NH*_*2*_
PTD4	YARAAARQARA*-NH*_*2*_
TAMRA-PTD4 TAMRA-PEG_3_-Tra(1,4)-C(O)-PTD4	TAMRA-PEG_3_-Tra(1,4)-C(O)-YARAAARQARA*-NH*_*2*_

### Synthesis of TAMRA-PEG_3_-N_3_

TAMRA-PEG_3_-N_3_ was synthesized in DCM/DMF solution (Cunningham et al. 2010). 6-carboxytetramethylrhodamine (TAMRA) was coupled to 1-amine-11-azido-3,6,9-trioxoundecan (N_3_-PEG_3_-NH_2_) using HATU with addition of DIPEA in DCM solution. The final product was lyophilized and purified. Next, it was analyzed with the use of RP-HPLC, and characterized by MALDI-TOF mass spectrometry.

### Synthesis of [Lys^7^(C(O)-Tra(1,4)-PEG_3_-TAMRA)]TP10 and TAMRA-PEG_3_-Tra(1,4)-C(O)-PTD4 with the use of “click chemistry”

The TAMRA labeled TP10 and PTD4 peptides, [Lys^7^(C(O)-Tra(1,4)-PEG_3_-TAMRA)]TP10 and TAMRA-PEG_3_-Tra(1,4)-C(O)-PTD4, were obtained with the use of “click chemistry”—a specific 1,3 dipolar Huisgen cycloaddition reaction (Colombo and Peretto 2008). The reaction of TAMRA-PEG_3_-N_3_ with [Lys^7^(Prop)]TP10 or Prop-PTD4 was carried out in water/*tert*-butanol medium in the presence of sodium ascorbate and CuSO_4_ as catalysts. The mixture was stirred at room temperature for 24 h. After the 1,2,3-triazole forming reaction had been completed, crude products were purified by RP-HPLC and characterized by analytical RP-HPLC and MALDI-TOF mass spectrometry.

### Preparation of test samples

There were ten stock solutions: 0.1 % cisplatin (3.33 × 10^−3^ M) and TP10 (10^−4^ M), TAMRA-TP10 (10^−4^ M), TP10 + cPt (10^−4^ M), TAMRA-TP10 + cPt (10^−4^ M), PTD4 (10^−4^ M), TAMRA-PTD4 (10^−4^ M), PTD4 + cPt (10^−4^ M), TAMRA-PTD4 + cPt (10^−4^ M), and TAMRA (10^−4^ M). The solvents were 0.9 % sodium chloride (NaCl).

The solutions of TP10 or TAMRA-TP10 + cPt (TP10 + cPt or TAMRA-TP10 + cPt) were prepared by mixing 1 ml of TAMRA-TP10 stock solution with 300-times diluted cisplatin stock solution (11.1 × 10^−6^ M), and the final ratio of TP10/cPt was 10:1. The solutions of PTD4 + cPt or TAMRA-PTD4 + cPt were obtained in the same way, using PTD4 or TAMRA-PTD4.

### Cell lines and culture conditions

Two human cancer lines—HeLa (cervical cancer) and OS 143B (osteosarcoma)—as well as one of the human non-cancer line—HEK293 (embryonic kidney)—were cultured in DMEM medium with 4.5 g glucose/l (Sigma-Aldrich, Germany) supplemented with 10 % fetal bovine serum (FBS) (Sigma-Aldrich, Germany) and 1 % PEN/STREP (Sigma-Aldrich, Germany). The second human non-cancer line—HEL299 (lung fibroblasts)—was cultured in EMEM medium (Sigma-Aldrich, Germany) supplemented in a similar manner as the abovementioned one, but with addition of 200 mM L-glutamine solution (Sigma-Aldrich, Germany). All cell lines were grown in a 5 % CO_2_ humidified atmosphere at 37 °C. The cell lines were obtained from the cell banks: HeLa (ATCC), HEL299 (ECACC), HEK293 (local cell bank), and OS 143B (local cell bank). The original lines were checked for the presence of mycoplasma.

### Cell viability assay

The MTT [3-(4,5-dimethylthiazol-2-yl)-2,5-diphenyl-2H-tetrazolium bromide] assay involved the following groups of experiments:

1. cPt, TAMRA-TP10

The compounds were investigated at the concentrations ranging from 0.1–40 μM on all tested cell lines after 24 h of incubation. The aim of this experiment was to find out the non-toxic concentrations in all cell lines.

Next, only non-toxic concentration of 1 μM was tested after 24, 48, and 72 h of incubation and the further groups were:

- carried out on four tested cell lines: non-cancer HEK293, HEL299 and cancer HeLa, OS143B

2) cPt, TAMRA-TP10, TAMRA-TP10 + cPt

- carried out on two tested cell lines: non-cancer HEL299 and cancer OS143B

3) cPt, TP10, TP10 + cPt, TAMRA
4) cPt, TAMRA-PTD4, TAMRA-PTD + cPt, PTD4, TAMRA-PTD4, TAMRA

MTT assay was based on the protocol described by Park et al. (1987). MTT (5 mg/ml) was dissolved in PBS, sterilized by filtration (0.22 μm Millipore® filter), and stored at 4 °C. Each tested cell line was seeded at a density of 10^4^ cells/well in a 96-well plate in 100 μl of culture medium and allowed to grow for 24 h before adding cPt, TAMRA, TP10, TP10 + cPt, TAMRA-TP10, TAMRA-TP10 + cPt and PTD4, PTD4 + cPt, TAMRA-PTD4, and TAMRA-PTD4 + cPt. Apart from the first experimental group, the abovementioned agents were only used at the final concentration of 1 μM. After treatment for 24, 48, and 72 h, the cells were washed twice in PBS and then 100 μl of 0.5 mg/ml of MTT in serum-free medium was added into each well. The incubation was continued for a further 3 h (time needed for MTT to become metabolized) at 37 °C. Formazan, a product of the former reaction, was dissolved in 100 μl of acidified isopropanol, and absorbance was measured at 570 nm using a DigiScan 340 microplate reader (Asys Hitech GmbH, Austria).

The results are presented as percentage of the control value, i.e., in relation to the untreated cells (100 %).

### Analysis of CPP ability to penetrate HEL299 and HeLa cell membranes

The ability of penetration of TP10 and PTD4 was possible to estimate due to their chemical binding with the orange fluorescent dye—TAMRA.

The procedure of preparing fluorescent preparations was as follows. Coverslips (∅20 mm) after washing with concentrated HCl and rinsing with 70 % ethanol were placed at the bottom of the 12-well plate. Cells were seeded at 10^5^ per 22 mm well (12-well plate) and left for 24 h. Thereafter, they were washed three times in PBS and then 200 μl of DMEM without FBS was added (cells starvation) and left for a further hour. Next, TAMRA-TP10, TAMRA-PTD4, TAMRA-TP10 + cPt, and TAMRA-PTD4 + cPt at a concentration of 10^−6^ M each were administered to the wells and the samples were incubated for 1 h.

The next step involved visualization of the cell nuclei (HEL299 and HeLa cells with the exception of the control—Fig. 6e, and TAMRA-PTD4—Fig. 6h) and membranes (HeLa cells excluding TAMRA-PTD4—Fig. 6h). Therefore, after washing the cells in PBS, they were treated with PKH67—a green dye for the membranes (according to the company protocol, Sigma-Aldrich, Germany) and finally with HO3342 (Hoechst 3342) dye—a blue one for the nuclei (Sigma-Aldrich, Germany), respectively. The latter dye was used in a final concentration of 1 μM. Then, the cells were fixed in 4 % formaldehyde solution for 15 min (diluted from 37 % stock, Sigma-Aldrich, Germany) and next, placed upside down on a Superfrost glass plate (Roth, Germany). One drop of the Vectashild mounting medium (Vector Laboratories, USA) was put between each mentioned glass plate and the coverslip. The edges of the coverslips were glued with a nail polish. The visualization was performed in a fluorescence microscope (Delta Optical IB-100, Delta Optical, Poland), and the fluorescence intensity emission for the dyes were as a follows: 562 nm—TAMRA, 352 nm—HO3342, and 502 nm—kit PKH67.

### Phase contrast microscopy

HEL299, HeLa, and OS 143B cells were seeded in a culture flask and cultured until gaining 70 % confluence. Next, the media were supplemented with cPt, TAMRA-TP10, and TAMRA-TP10 + cPt in concentrations of 10^−6^ M each. The incubation was continued for 5 days, and the supplemented medium was changed for a fresh one every second day. Cells were observed in inverted phase contrast microscope Delta Optical IB-100 equipped with an optical camera HDCE-50B at ×20 objective (Delta Optical, Poland).

### Statistics

The data of MTT test were expressed as a mean of three independent experiments conducted in triplicates for each concentration. On this basis, standard error of measurement (SEM) was calculated. The data of fluorescent microscopy included minimum 3-time repeated experiments and those of contrast phase microscopy at least three times. Data were analyzed by Friedman ANOVA and Kendall Concordance using STATISTICA version 9.1 data analysis software system, and *p* < 0.05 was assumed to be statistically significant (StatSoft, Inc., 2010, www.statsoft.com.

## Results

### The effect of different concentrations of TAMRA-TP10 on non-cancer (HEK293, HEL299) and cancer (HeLa, OS143B) cell lines

**Fig. 1:**
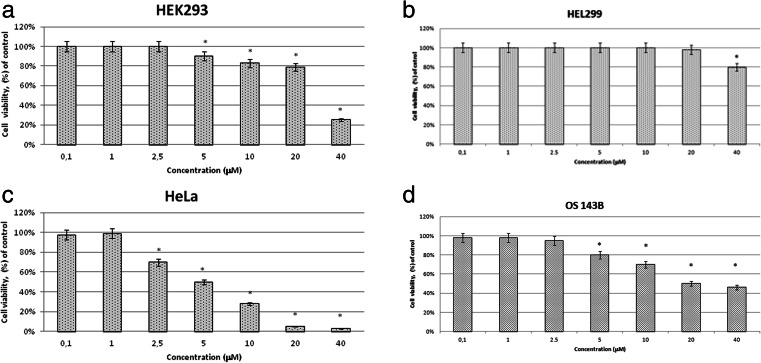
Viability of **a** HEK293, **b** HEL299, **c** HeLa, and**d** OS143B cell lines measured with MTT assay after 24 h incubation with TAMRA-TP10. All data represent the mean ± SEM of five independent experiments conducted in triplicates. Differences in viabilities were statistically significant (marked by *asterisk*) as compared to the control (*p* < 0.05)

All tested cell lines were incubated with TAMRA-TP10 at concentrations in the range of 0.1–40 μM. The experiments were performed in order to select TAMRA-TP10 concentration for further research. The criterion of the selection was its lack of toxicity in each cell line (Fig. 1).

Figure 1a, b shows the results of viability of non-cancer (HEK293 and HEL299) and Fig. 1c, d shows cancer (HeLa, OS143B) cell lines after 24 h incubation with TAMRA-TP10. As it turned out, the concentration of 1 μM was not toxic in all tested cell lines, and cell viability remained at the control level (cells without any agent). The concentrations above 1 μM (2.5–40 μM) of TAMRA-TP10 reduced the cell number especially in the cancer cell lines. For example, in the HeLa one, there was a progressive concentration-dependent fall in cell viability so that the inhibition of cell viability approached almost 100 % at the concentration of 40 μM.

This concentration of TAMRA-TP10 affected also both healthy cell lines (about 80 % or 20 % drops of viability in HEK293 or HEL299 lines, respectively).

### The effect of different concentrations of cPt on non-cancer (HEK293, HEL299) and cancer (HeLa, OS143B) cell lines

**Fig. 2:**
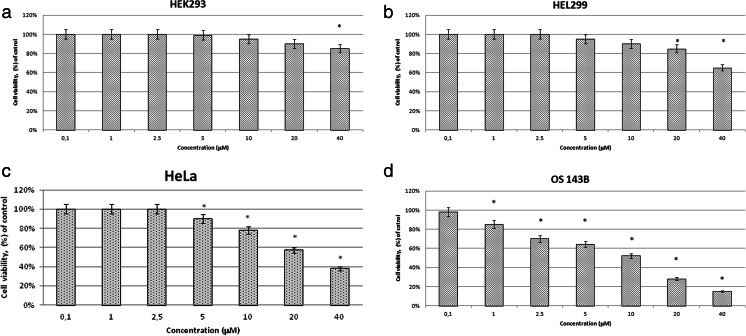
Viability of **a** HEK293, **b** HEL299, **c** HeLa, and **d** OS143B cell lines measured with MTT assay after 24-h incubation with cPt. All data represent the mean ± SEM of five independent experiments conducted in triplicates. Differences in viabilities were statistically significant (marked by *asterisk*) as compared to the control (*p* < 0.05)

cPt at the concentration of 1 μM did not affect cell viability in non-cancer cell lines (Fig. 2a, b). Considering the cancer cell lines, there was a small, although a significant, drop of survival in the case of OS143B one (Fig. 2d).

The higher concentrations of the drug produced an evident inhibition of cell viability in the cancer cell lines (Fig. 2c, d). High sensitivity to this treatment showed OS143B line (Fig. 2d) in which the decreases in cell viability started already from the concentration of 1 μM and culminated at the one of 40 μM (ca. 80 %). On the other hand, the effect of cPt concentrations above 1 μM on non-cancer cell lines was much smaller (Fig. 2a, b) and appeared particularly at the highest concentration (about 15 or 35 %).

### Analysis of the action of TAMRA-TP10 + cPt and TP10 + cPt on non-cancer (HEK293, HEL299) and cancer (HeLa, OS143B) cell lines

**Fig. 3:**
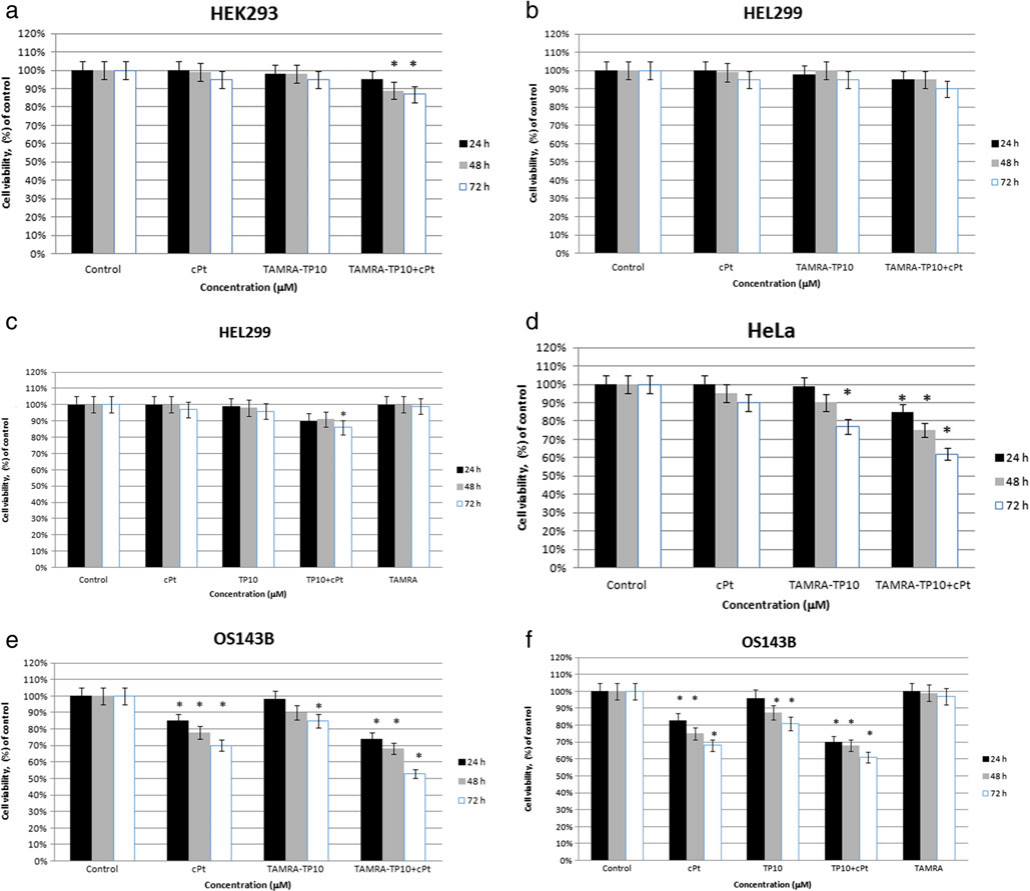
Viability of **a** HEK293, **b** HEL299, **c** HEL299, **d** HeLa, **e** OS143B, and **f** OS143B cells measured with MTT assay after 24 h (*black*), 48 h (*gray*), and 72 h (*white*) incubation with 1 μM of cPt, *TAMRA-*TP10, TP10, *TAMRA-*TP10 + cPt, TP10 + cPt, and *TAMRA*. All data represent the mean ± SEM of five independent experiments conducted in triplicates for each compounds. Differences in viabilities were statistically significant (marked by *asterisk*) as compared with control (*p* < 0.05)

Figure 3 presents the effect of 24-, 48-, and 72-h incubation with TAMRA-TP10 + cPt (1 μM) and its components—cPt (1 μM) or TAMRA-TP10 (1 μM)—on HEK293 (Fig. 3a), HEL299 (Fig. 3b), HeLa (Fig. 3d), and OS143B (Fig. 3e) cell lines. Furthermore, this figure includes also the results obtained after treatment of two lines (HEL299—Fig. 3c and OS143B—Fig. 3f) with the same agents, but without TAMRA (TP10, TP10 + cPt), and with TAMRA alone.

All tested probes were compared to a sample without any compounds, used as a negative control.

The action of TAMRA-TP10 + cPt on healthy cell viability (Fig. 3a, b) was rather minimal and became most evident after 72 h. The changes after cPt or TAMRA-TP10 were even smaller irrespective of the time of exposition.

The response of the cancer cell lines to the treatment with TAMRA-TP10 + cPt. Figure 3d, e characterizes time-progressive decreases in cell survival culminating in the final drops of about 40 % (HeLa cells) and 50 % (OS143B cells). All of them were statistically significant in comparison to the controls (100 %). There was a difference in sensitivity of each cancer line to cPt (Fig. 3d, e). A more noticeable effect occurred on OS143B cells (a progressive fall with a max. of 30 % after 72 h). TAMRA-TP10 affected similarly viability of both cancer cell types, and the falls achieved statistical significance after 72 h (Fig. 3d and Fig. 3e).

To avoid the impact of TAMRA itself, the effect of the compounds in question (TP10 + cPt or TP10) without the dye was analyzed on one non-cancer (Fig. 3c) and one cancer (Fig. 3f) line.

It turned out that their effects on both cell lines were comparable to those noticed after the complex with this dye or TP10 with it (Fig. 3c, f). What is more, TAMRA dye did not affect the viability of both cell lines (Fig. 3c, f). The lack of cytotoxicity of this fluorescent dye is congruent with earlier data considering its usage safety (Alford et al. 2009).

### Analysis of the action of TAMRA-PTD4 + cPt and PTD4 + cPt on non-cancer (HEL299) and cancer (OS143B) cell lines

**Fig. 4:**
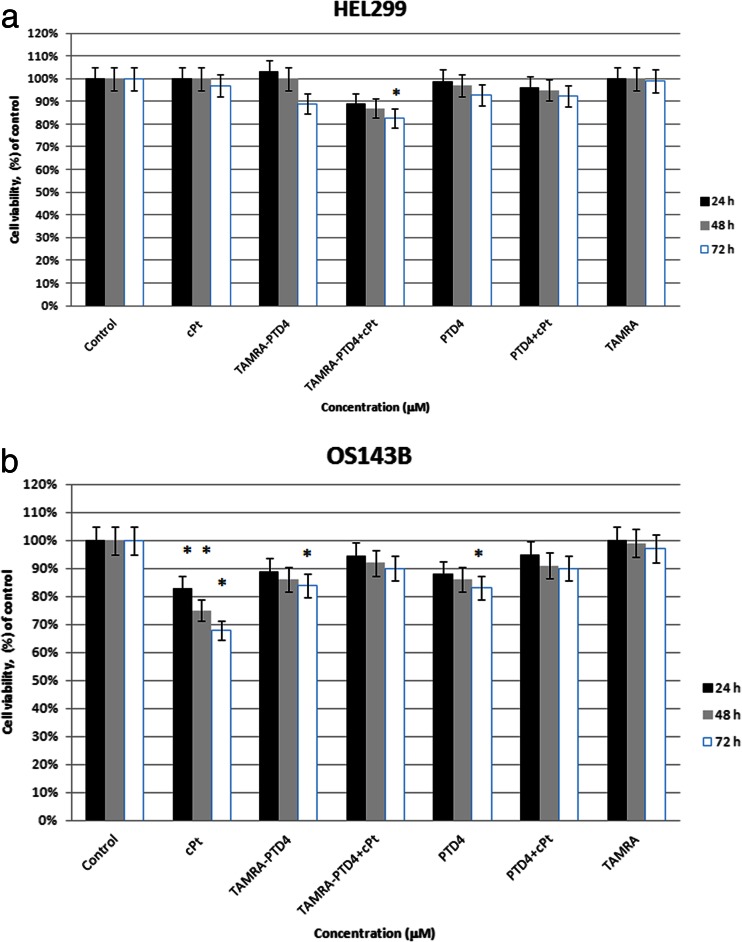
Viability of **a** HEL299 and **b** OS143B cells measured with MTT assay after 24 h (*black*), 48 h (*gray*), and 72 h (*white*) incubation with 1 μM of cPt, *TAMRA-*PTD4, *TAMRA-*PTD4 + cPt, PTD4, PTD4 + cPt, and *TAMRA*. All data represent the mean ± SEM of three independent experiments conducted in triplicates for each compounds. Differences in viabilities were statistically significant (marked by *asterisk*) as compared with control (*p* < 0.05)

PTD4 + cPt (with TAMRA or without) as well as PTD4 (with TAMRA or without) did not indicate a statistically significant effect on the healthy cell viability (Fig. 4a). A lack of statistical significance was also observed in PTD4 + cPt (with TAMRA or without)-treated OS143B cells (Fig. 4b). In the case of PTD4 (with TAMRA or without), a statistically significant effect (15 % decrease in cell viability) appeared only after 72 h of incubation.

### Localization of TAMRA-TP10, TAMRA-TP10 + cPt, TAMRA-PTD4 and TAMRA-PTD4 + cPt in HEL299 and HeLa cells

The ability of crossing the cell membrane by the compounds in question as well as their localization in the cell was verified by fluorescence microscopy. HEL299 and HeLa cells were incubated with *TAMRA*-TP10, *TAMRA*-TP10 + cPt, *TAMRA*-PTD4, and *TAMRA*-PTD4 + cPt.

**Fig. 5:**
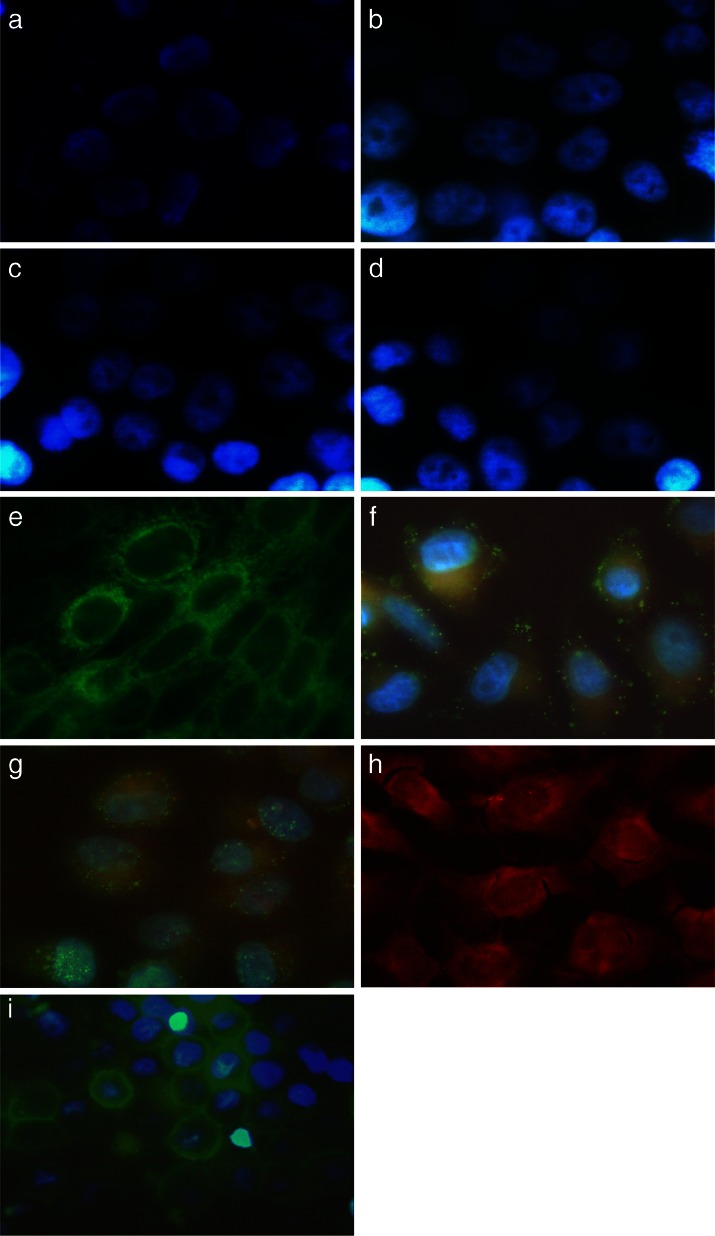
Transporting ability of *TAMRA-*TP10, *TAMRA-*TP10 + cPt, *TAMRA-*PTD4, and *TAMRA-*PTD4 + cPt through the membrane of HEL299 or HeLa cells and intracellular localization visible in fluorescent microscopy (×100 objective except for **i**—×40). HEL299: **a** *TAMRA-*TP10 (*orange*, *blue dyes*), **b** *TAMRA-*TP10 + cPt (*orange*, *blue dyes*), **c** *TAMRA-*PTD4 (*orange*, *blue dyes*), and **d** *TAMRA-*PTD4 + cPt (*orange*, *blue dyes*). HeLa: **e** control (*green dye*), f *TAMRA-*TP10 (*orange*, *green*, *blue dyes*), **g** *TAMRA-*TP10 + cPt (*orange*, *green*, *blue dyes*), **h** *TAMRA-*PTD4 (*orange dye*) and **i** *TAMRA-*PTD4 + cPt (*orange*, *green*, *blue dyes*). Figures are representatives of minimum three independent experiments

Figure 5a–d presents the picture of HEL299 cells after exposition to the tested compounds. As can be seen, there is no internalization of the orange dye inside the cells. This observation may be evidence for a lack of penetrating ability through the healthy cell membranes of both tested CPPs.

Figure 5e is a control picture of cancer HeLa cells without any compounds. In this picture, the cell membranes are clearly marked green for illustration of the their morphology. HeLa cells incubated with *TAMRA-*TP10 are presented in the next picture (Fig. 5f), and it shows the presence of this agent inside the cells (orange color). Figure 5g visualizes cancer cells after incubation with *TAMRA-*TP10 + cPt. Again, the orange color is visible inside the cell. For a better visualization of the compounds in question, the cell membranes and nuclei were stained green or blue, respectively.

Additionally, the penetrating ability of another CPP, i.e., *TAMRA-*PTD4, was tested on HeLa cell line. This CPP, being able to cross the cell membrane, is visible inside the cells (Fig. 5h). On the other hand, if *TAMRA-*PTD4 was used in the complex with cPt (Fig. 5i), there is no internalization of the orange dye, which suggests that PTD4 in this case lost its penetrating ability.

Also, TAMRA alone does not penetrate the cellular membrane and it is localized outside the cell (not shown).

### Long-term effects of TAMRA-TP10 + cPt

**Fig. 6:**
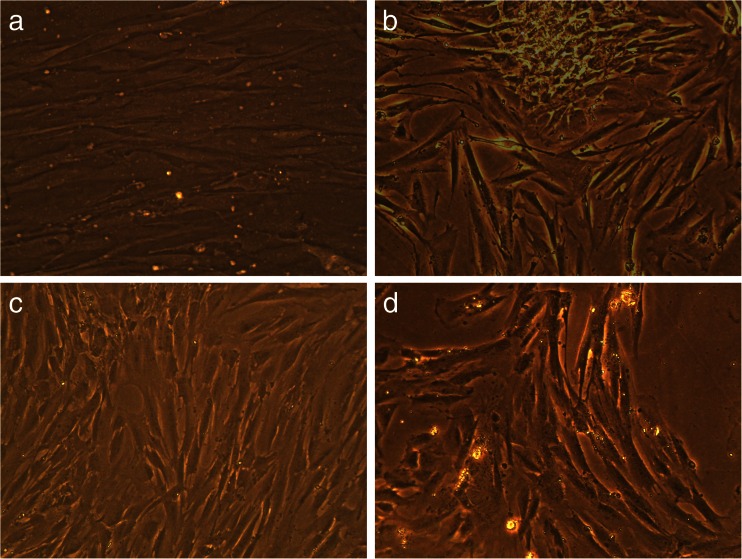
Long-term toxicity (after 5-day incubation) of the compounds in HEL299 cells, **a** untreated cells, **b** treated with cPt, **c** treated with TAMRA-TP10, and **d** treated with TAMRA-TP10 + cPt. Figures are representatives of minimum three independent experiments (×20 objective)

**Fig. 7:**
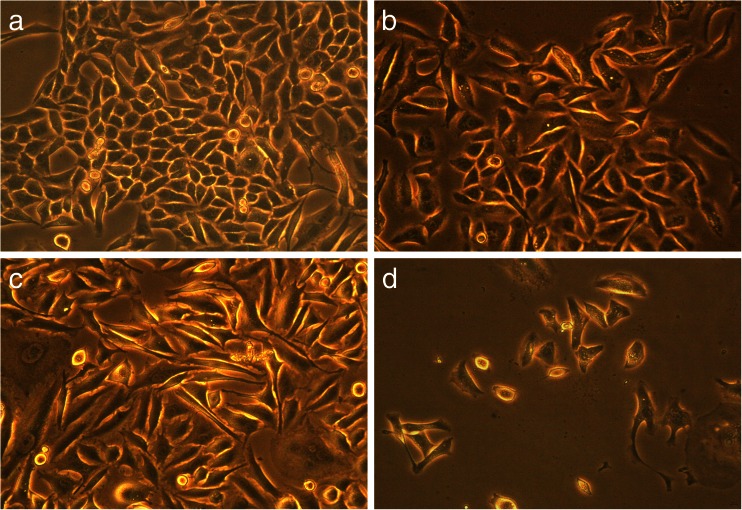
Long-term toxicity (after 5-day incubation) of the compounds in HeLa cells, **a** untreated cells, **b** treated with cPt, **c** treated with TAMRA-TP10 and **d** treated with TAMRA-TP10 + cPt. Figures are representatives of minimum three independent experiments (×20 objective)

**Fig. 8:**
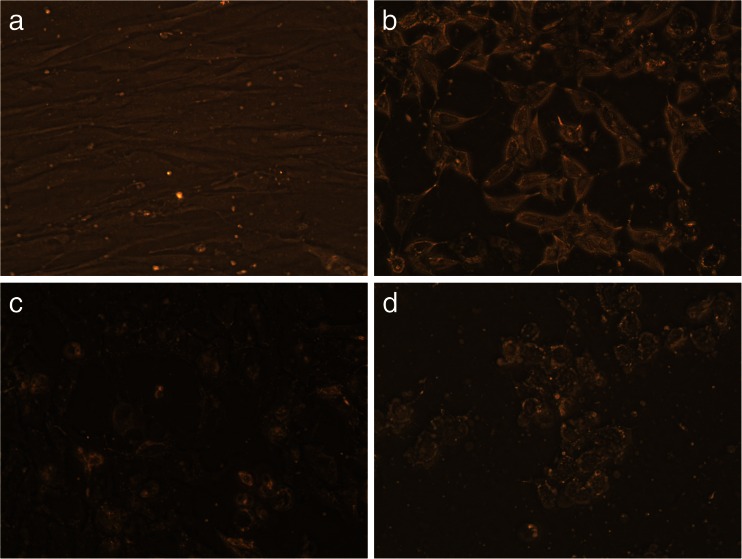
Long-term toxicity (after 5-day incubation) of the compounds in OS143B cells, **a** untreated cells, **b** treated with cPt, **c** treated with TAMRA-TP10, and **d** treated with TAMRA-TP10 + cPt. Figures are representatives of minimum three independent experiments (×20 objective)

Long-term effects of *TAMRA-*TP10 + cPt, cPt, and *TAMRA-*TP10 were checked on one non-cancer cell line (HEL299) and two cancer (HeLa, OS143B) cell lines for 5 days. The results of this experiments are presented in the pictures bellow (Figs. 6, 7, and 8).

The first of them (Fig. 6a–d) presents results obtained after the usage of the tested compounds on non-cancer cells. A rather small decrease in the number of cells (without their morphological changes) is only visible after long-term exposition to *TAMRA-*TP10 + cPt (Fig. 6d).

Figure 7 and Fig. 8 show changes in the number and morphology of the cells induced by the compounds in question in the cancer cell lines. The most dramatic effect (complete or almost complete killing of the cells) is noticed after treatment with TAMRA-TP10 + cPt (Fig. 7d and Fig. 8d). Also, cPt (Fig. 7b and Fig. 8b) as well as TAMRA-TP10 (Fig. 7c and Fig. 8c) affect these cells with a resultant reduction of their population, change in morphology, and loss of the connections between them.

Since the results obtained after the tested compounds with TAMRA or without it are almost the same, the discussion of the results in the next section will be carried out with the omission of the dye presence in the given agent.

## Discussion

TP10 + cPt—investigated in this study with respect to anticancer activity—represents a non-covalent carrier-cargo complex with a metal-affinity-based linkage. This non-covalent strategy, except for its drawbacks, is more advantageous over covalent conjugation with regard to its simplicity, versatility of cargo composition, lowered concentrations needed to induce biological response, and easy intracellular cleavage to non-toxic metabolites (Lindgren et al. 2004; Durzyńska et al. 2015).

As the presented here experiments have demonstrated, this complex produced a more pronounced anticancer effect on HeLa and OS143B cells in comparison with that observed after incubation with each single compound, i.e., the transportan or the anticancer drug. This statement could be made on the basis of the MTT test, which has shown that TP10 + cPt reduced progressively the number of cancer cells with a maximum between 40 and 50 % after 72 h. At the same time, cPt or TP10 applied separately produced a much smaller effect, i.e., a 10–30 % or a 15 % reduction in cell survival, respectively.

In the light of the presented data, the following assumptions could be made. The fact that TP10 enhanced the effect of cPt (if given in the form of a complex) may be a result of interactions which may occur between TP10 and cPt. These interactions may have a pharmacokinetic or pharmacodynamic dimension.

The possible pharmacokinetic interactions may concern cellular bioavailability. TP10 could intensify the transport of cPt through the cellular membrane and thereby increase the internalization of this anticancer drug. Similar observations have been made by others with the usage of CPP conjugates with such small chemotherapeutic anticancer drugs as doxorubicin, methotrexate, or paclitaxel (Durzyńska et al. 2015; Rousselle et al. 2000; Lindgren et al. 2006). All of them indicated improved anticancer activity (in comparison with the free drugs) which was attributed to a more efficacious cell internalization. What is more, the studies have been carried out on different cancer cell lines as MDA-MB-231 and K562 (Saar et al. 2005).

The issue of how CPPs deliver molecules to the cell is still a matter of controversy. There is also no consensus considering cell uptake of transportans and its derivatives. Most of the experimental data provide evidence that their translocation occurs via energy-independent route with the usage of versatile pathways, as for example different types of endocytosis, non-endocytic processes, like pore formation in the membranes, formation of inverted micelles, membrane fusion, and rupture (Islam et al. 2014; Choi and David 2014; Durzyńska et al. 2015). In the case of TP10, it reaches probably the cell interior by concentration-dependent non-endocytotic pathways, which was indicated in experiments with the use of a single giant unilamellar vesicle. They revealed continuous translocation across the lipid membrane at the lower concentrations of TP10 and formation of pores at the higher ones (Islam et al. 2014).

In the context of this study, it is worth stressing that among the investigated CPPs, only TP10 was able to improve the action of cisplatin. As the results of the MTT assay showed, such phenomenon did not occur when another CPP, i.e., PTD4 was used in the form of a complex with this anticancer drug. The effect of PTD4 + cPt was even smaller than that observed after cPt itself.

PTD4 is known for the delivery of proteins across the cellular membrane in a very efficacious way (Ho et al. 2001). Also, the fluorescent microscopy experiment carried out in this study indicated that TAMRA-PTD4 gained access to the intracellular compartment. However, such an effect was not present after incubation with TAMRA-PTD4 + cPt. Apparently, in the case of this complex, PTD4 lost the transporting activity probably due to alkylation of its chain by cPt. This did not happen with the TAMRA-TP10 + cPt complex because the fluorescent dye became visible in the interior of the cells.

The next issue to be discussed will concern the possible pharmacodynamic interactions between TP10 and cPt which may serve as an explanation for the enhanced anticancer activity of TP10 + cPt. Of course, this kind of interaction assumes such an activity of the CPP itself. In fact, a small anticancer activity of TP10 which has been found in both cancer cell lines (MTT assay) could be additive to the one performed by cPt.

The anticancer activity of TP10 does not seem to be very surprising as certain CPPs together with TP10, apart from the ability to translocate the cargo, are known for other activities, including the antitumor one (Reddy et al. 2004; Powers and Hancock 2003; Leuschner and Hansel 2004). Although their mechanism of the anticancer activity is not fully understood, it may at least partly be due to the membrane perturbation (induction of membrane leakage) and/or to binding to the intracellular targets (Saar et al. 2005; Mader et al. 2005). Considering the latter one, there is not much data available. Among them, that provided by Mader et al. (2005), although concerning another CPP (lactoferricin), seems to be attractive. In this report, the site of action was attributed to the mitochondria in which the abovementioned CPP triggered an apoptotic pathway.

It was also found that TP10 and PTD4 had no impact on cell viability in non-cancer (HEK293 and/or HEL299) cell lines. An explanation for this experimental fact may be a different access of the tested compounds to healthy or cancer cells. Such a difference in cellular access was visualized by fluorescent microscopy, which showed a lack of internalization of TP10 and PTD4 in the former ones. Thus, it may be assumed that these compounds indicate a kind of selectivity to the cancer cell lines. A similar phenomenon was reported by Song et al. 2011 and Saar et al. 2005 who also tested TP10, but on other cell lines (non-cancer: NIH-3T3, AEC and cancer: K562, MDA-MB-231). As the experiments performed in this study indicated, the feature of selectivity could also be ascribed to TP10 + cPt.

It is not known which factors provide the specificity of cationic peptides for the cancer cell membranes. However, certain features of these membranes have been pointed out as possible reasons, e.g., different membrane composition and fluidity, more negative charge, higher transmembrane potential, and increased level of acidic components on the surface (Leuschner and Hansel 2004; Papo and Shai 2003). The membranes of cancer cells—versus those of normal ones (which consist of neutral zwitterionic phospholipids and sterols)—carrying a net negative charge (due to increased expression of anionic molecules, i.e., phosphatidylserine, heparan sulfates) (Hoskin and Ramamoorthy 2008; Schweizer 2009) could electrostatically attract a CPP. Probably, such phenomenon also occurs if TP10 is present in the environment. In this context, it appears to be understandable why this peptide was preferentially bound to cancer cell membranes and gained easier access to the cancer cells in comparison to the healthy ones.

The discussed above possible pharmacokinetic and pharmacodynamic interactions between TP10 and cPt could be predictable to some extent taking into account the unique properties of this transportan. These properties had also an impact on its choice in the case of this study. Among them, a special attention should be paid to the presence of lysine (Lys) in the chemical structure of TP10. Its role seems to be complex. As has been indicated, a reactive amino group at Lys is a suitable handle to connect this CPP to such cargos as hydrophilic macromolecules (Pooga et al. 1998), including cPt. Furthermore, the number of Lys molecules enhances the transportan’s amphipathicity which is an important parameter that affects translocation of CPPs (Alves et al. 2008; Deshayes et al. 2004; Song et al. 2011).

The last section of the discussion is devoted to the 5-day effect of the complex (TP10 + cPt) and its individual components on one non-cancer and two cancer cell lines. This long-term effect has been estimated on the basis of inverted phase contrast microscopic images. As compared with TP10 or cPt, the most potent action on the cancer cells has become visible if the complex was applied. Thus, the presented here microscopic images may support the results obtained in the MTT test as well as the conclusions drawn on their basis.

In summary, this study has demonstrated an improved anticancer effect of TP10 + cPt complex in comparison with that produced by cPt. This effect could result from pharmacokinetic and/or pharmacodynamic interactions between the complex’s components. Additionally, TP10 + cPt was relatively safe for the non-cancer cell lines. Although, these conclusions have been drawn only on the basis of a qualitative analysis, they may serve as grounds of further more detailed research which would open new clinical prospects for this old anticancer drug.

## Abbreviations

AEC: SV40-immortalized aortic endothelial cells
ATCC: American Type Culture Collection
Boc: *tert-*butyloxycarbonyl
CPP: cell penetrating peptides
cPt: cisplatin
DCM: dichloromethane
DIPEA: *N*,*N*-diisopropylethylamine
DMF: *N*,*N*-dimethylformoamide
ECACC: European Collection of Cell Cultures
Fmoc: 9-fluorenylmethoxycarbonyl
Gal: galanin
HATU: 2-(1*H*-7-azabenzo-triazole-1-yl)-1,1,3,3-tetramethylouronium hexafluorophosphate
HOBt: *N*-hydroxybenzotriazole
ivDde: 1-[4,4-dimethyl-2,6-dioxocyclohex-1-ylidene)-3-methylbutyl
K562: erythroleukemia
MALDI-TOF: matrix-assisted laser desorption/ionization - time of flight
Mas: mastoparan
MDA-MB-231: breast cancer
NIH3T3: mouse embryonic fibroblast
NMP: *N*-methyl-2-pyrrolidone
PEG_3_: 3,6,9-trioxoundecan
PEN/STREP: penicillin/streptomycin
Prop: propynyl
PTD4: Protein transduction domain 4
RP-HPLC: reverse phase high-performance liquid chromatography
SPPS: solid phase peptide synthesis
TAMRA: 6-carboxytetramethylrhodamine
Tat: Trans-activator of transcription
TBTU: *O*-(benzotriazole-1-yl)-1,1,3,3-tetramethyluronium tetrafluoroborate
TFA: trifluoroacetic acid
TIPS: triisopropylsilane
TP10: transportan 10
Tra: 1,2,3-triazole
